# Electrical terahertz modulator based on photo-excited ferroelectric superlattice

**DOI:** 10.1038/s41598-018-21095-5

**Published:** 2018-02-08

**Authors:** Jie Ji, Siyan Zhou, Jingcheng Zhang, Furi Ling, Jianquan Yao

**Affiliations:** 1 Wuhan National Laboratory for Optoelectronics, Huazhong University of Science and Technology, Wuhan, 430074 China; 2 School of Optical and Electronic Information, Huazhong University of Science and Technology, Wuhan, 430074 China; 3 College of Precision Instrument and Optoelectronics Engineering, Tianjin University, Tianjin, 300072 China

## Abstract

The transmission and dielectric spectra of ferroelectric STO/PT superlattice on Si substrate under simultaneous external optical and electric field were investigated and compared at room temperature. Results found that when with an optical field, the electric field realized an effective modulation on the transmission, which displayed a diode property. In addition, a comprehensive model combined with Debye relaxation and Lorentz model was used to analyze the dielectric spectra, variation of the soft mode with external field was put emphasis on exploring.

## Introduction

Terahertz (THz) electromagnetic waves lie in the frequency range 0.1–10 THz, a scientifically rich frequency band that is of unique value for chemical identification and characterization of the materials, such as electronic and vibrational properties^1,2^. In recent years, moderate progress has been made in THz generation and detection, such as THz quantum cascade lasers and time-domain THz spectrometer^3–5^. These technologies may contribute to a multitude of terahertz applications that are currently under investigation globally^6^. However, greater efforts are required to effectively manipulate terahertz radiation. There are comparatively few methods to electrically modulate or switch a THz wave, a critical requirement in many proposed THz communication systems^7–10^. Terahertz modulator based on semiconductor was the most common one as THz waves can be absorbed by a conductor, and the basic way to modulate THz waves is to tune conductivity of a semiconductor with an external voltage signal or a pumping light^11,12^. For example, Berardi Sensale-Rodriguez *et al*. effectively tuned transmission of THz wave via electrically tuning the Fermi energy of graphene and the density of states available for intraband transitions^13^. Hou-Tong Chen *et al*. demonstrated an active metamaterial device capable of manipulation of THz radiation^14^. The metamaterial array and substrate together effectively form a Schottky diode, which enables modulation of THz transmission. As another traditional material, ferroelectrics are well known for their essential characteristic: spontaneous polarization. The combination of ferroelectric thin film and semiconductor provides large potentials for the development of integrated devices, such as memories, detectors and optoelectronics^15–18^.

In recent years, ferroelectric-dielectric heterostructures have been the focus of experimental and theoretical attentions due to their polarization enhancement and other improved properties over single-component thin film^19–21^. They offer a unique opportunity to investigate the response of such nanodomain to uniform applied fields and in addition to engineer domain structures which enhance the functional properties of these artificial materials^22^. PbTiO_3_ (PT) and SrTiO_3_ (STO) are both a displacive ferroelectric with the ABO_3_ perovskite-type structure, and PT is in ferroelectric phase at room temperature while STO is in paraelectric phase, indicating that STO has no spontaneous polarization^23,24^. The photon structure of PT and STO has been extensively studied by theoretical calculation and experiments, including neutron scattering, Raman scattering, and far-infrared spectroscopy, respectively^25–28^. Low-frequency dielectric characteristics are mostly dependent on soft mode and relaxation^29^. Most studies on the dielectric properties and the soft mode behavior of ferroelectric thin films have been conducted with external electrical or temperature or optical fields, separately^30–32^. Researches about analyzing dielectric property of ferroelectric thin film under multiple external field have rarely been seen, especially under the joint effect of electric and optical field. In this manuscript, transmission and dielectric properties of the ferroelectric STO/PT superlattice on Si substrate were investigated under external optical and electric field simultaneously by THz spectrometer; a comprehensive model combined with Debye relaxation and Lorentz model was used to analyze the dielectric spectra.

**Figure 1:**
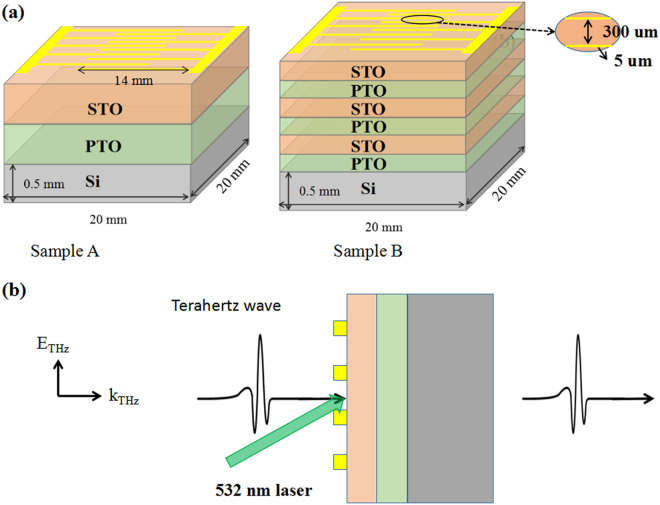
(**a**) Schematic diagram of two heterostructures ferroelectric superlattices on Si substrate. (**b**) Orientation of the electric field vector and wave vector of the incident terahertz radiation.

In this experiment, ferroelectric superlattices consisting of PT thin films and STO thin films were prepared by a radio frequency magnetron sputtering apparatus. (100)-oriented 20 × 20 × 0.5 mm^3^ Si crystal was used as a substrate. Interdigital electrodes including 12 pairs of gold stripes with 5 microns wide were prepared by lift-off photolithography and subsequent deposition of Cr film as the adhesion layer and Au film by electron beam evaporation. The gap between each couple of stripes was 300 microns wide. The two samples were prepared as a multilayer structure with interdigital electrodes as shown Fig. 1(a).

The transmission and dielectric properties of the ferroelectric superlattices on Si substrate in THz frequency domain were obtained by a THz time-domain spectrometer (THz-TDS) system produced by Zomega Terahertz Corporation (USA). An all-solid-state green continuous wave laser (center wavelength, 532 nm) was employed for an external optical pumping in this experiment, the green light was obliquely incident upon the film at 60°. In addition, a direct current voltage was also applied to provide an electric field. In this experiment, the wave vector of the terahertz radiation was perpendicular to the plane of the thin film structure, and the electrical field of the terahertz radiation was also perpendicular to the electrode fingers, as shown in Fig. 1(b).

## Results and Discussion

**Figure 2:**
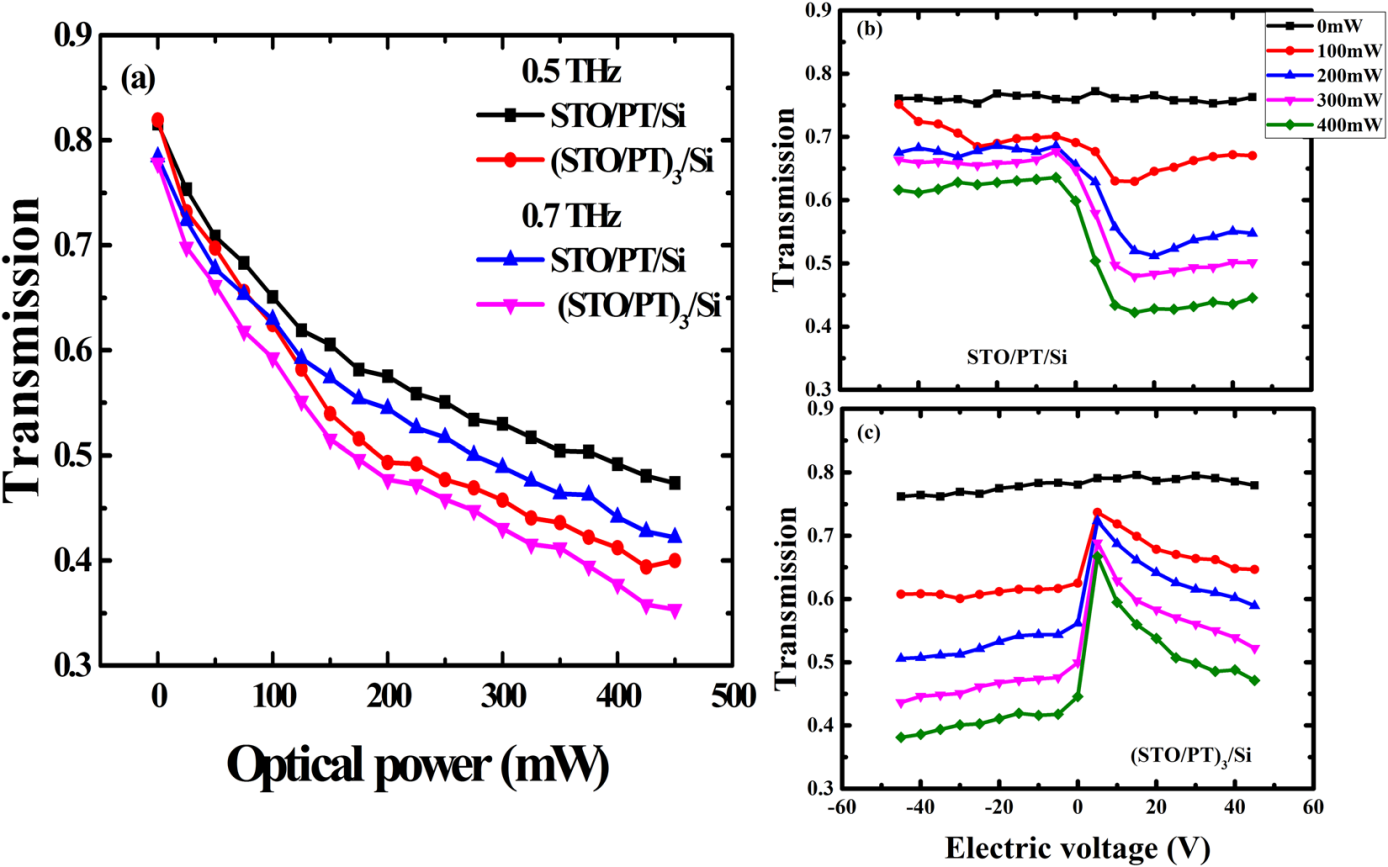
Transmission of the two samples. (**a**) Transmission of the two samples without interdigital electrodes as function of the external optical pumped powers. (**b**) Transmission of the sample A with the electric voltages at optical powers P = 0, 100, 200, 300, 400 mW, respectively. (**c**) Transmission of the sample B with the electric voltages at optical powers P = 0, 100, 200, 300, 400 mW, respectively.

Figure 2(a) compared transmission properties of the two sample with the external optical power at 0.5 THz and 0.7 THz, respectively. When the samples were photo excited, we observed transmission modulation, which was defined as ${\rm{M}}=|\frac{{{t}}_{{\rm{\max }}}-{t}_{{\rm{\min }}}}{{t}_{{\rm{\min }}}}|\times 100 \%$. The increase in the optical power caused a visible change, corresponding to a modulation depth of 46.3% and 56.2%, respectively. In addition, it could be observed that the sample B was more sensitive to optical powers as there were more interfaces existed in it. Related researches were investigated previously. For ferroelectric thin film, more strain induced by the interfaces would increase the sensitivity over the optical power^33^. Figure 2(b) and (c) displayed transmission properties of the two samples as function of applied electric voltages with different optical pump powers. When without optical excitation, the applied bias voltage did not cause a significant change in the terahertz transmission of the two samples. However, once the sample was illuminated by the green laser, transmission characteristics of the two sample were varied differently. To be specific, when sample A was at negative bias, the increase in voltage contributed to a negligible change in the transmission. On the contrary, a drastic decrease in the terahertz transmission was observed with the increase in positive bias voltage from 0 V to 20 V. Besides, sample B at bias no matter it is positive or negative, increments in voltages both resulted in a decrease in the transmission. The positive voltage displayed a higher modulation depth. When the optical power was tuned to 400 mW, electric modulation depth of the sample A and sample B reached to 48.7% and 78.5%, respectively.

In such a ferroelectric thin film–silicon hybrid structure, we attributed the observed transmission characteristics to the variation in dielectric constant of ferroelectric layer due to the hybrid interfaces and excited carrier density. To acquire further understanding of the transmission characteristics, we now discuss dielectric properties of the ferroelectrics under the hybrid fields.

**Figure 3:**
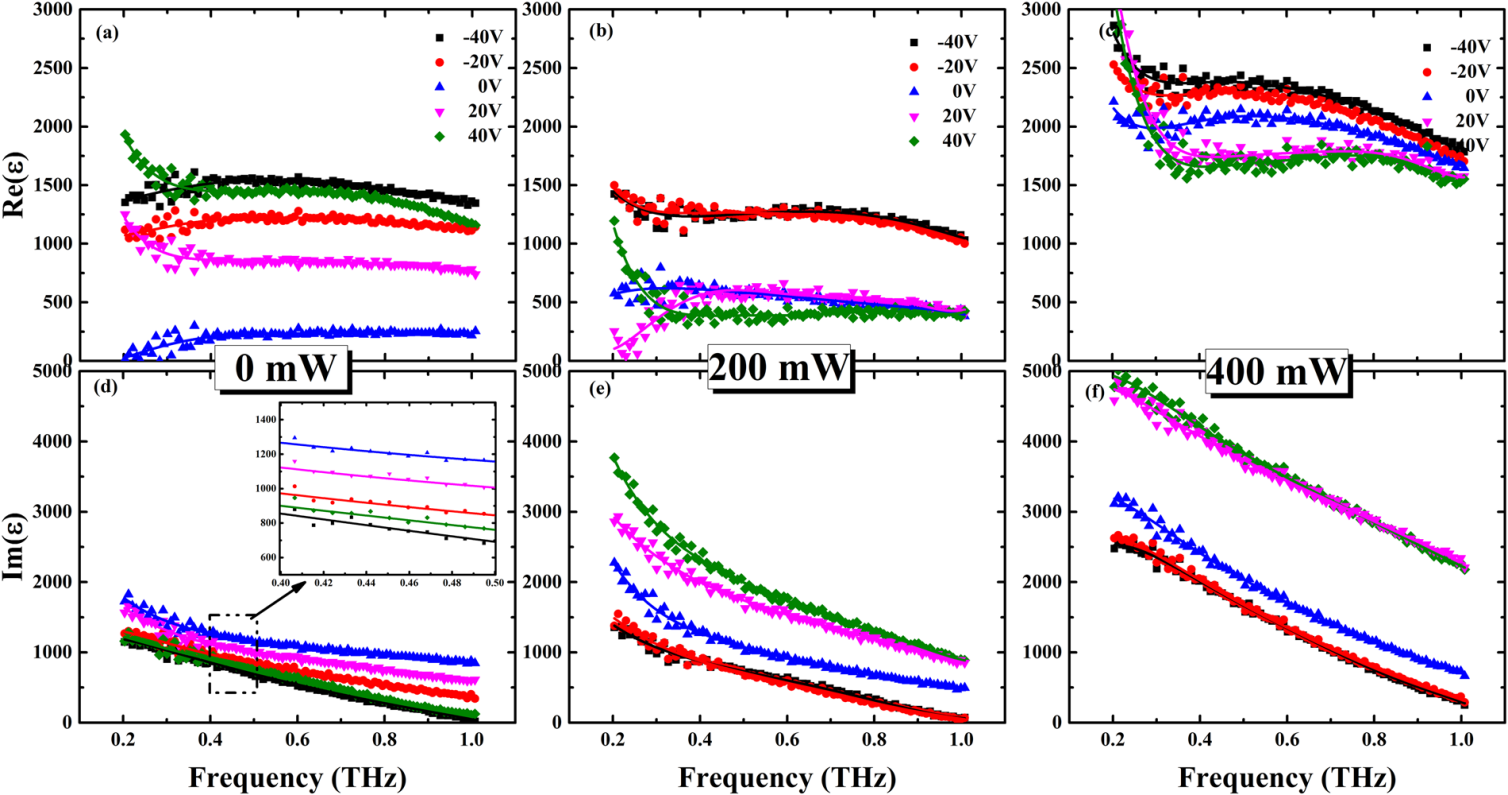
Measured permittivity of ferroelectric superlattice in the sample A. (**a**,**b** and **c**) Were measured real parts of dielectric constant biased at −40 V, −20 V, 0 V, 20 V, 40 V with the optical pump power of 0, 200 and 400 mW, respectively. (**d**,**e** and **f**) Were measured imaginary parts of dielectric constant corresponding to (**a**,**b** and **c**), respectively.

**Figure 4:**
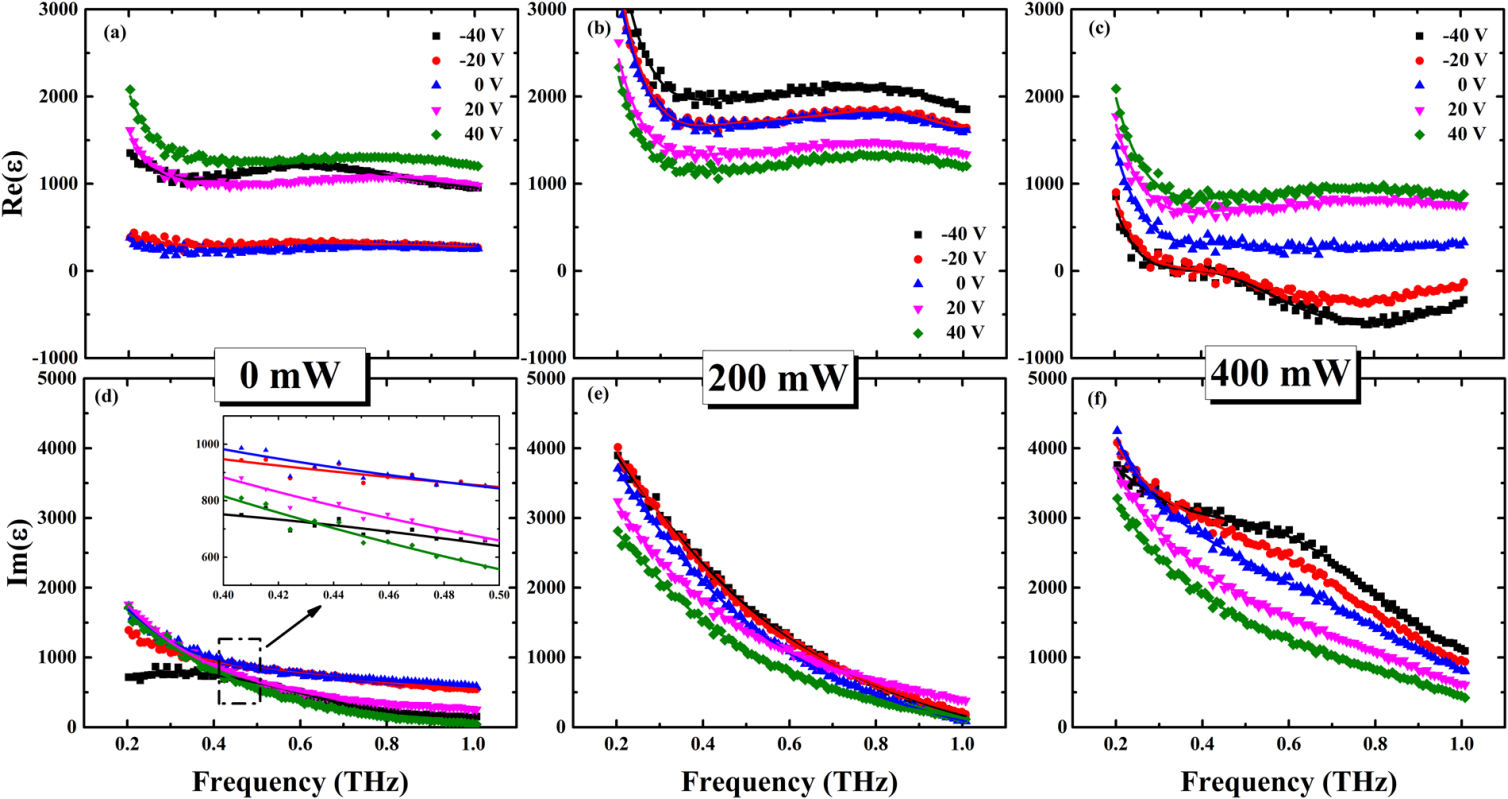
Measured permittivity of ferroelectric superlattice in the sample B. (**a**,**b** and **c**) Were measured real parts of dielectric constant biased at −40V, −20V, 0 V, 20 V, 40 V with the optical pump power of 0, 200 and 400 mW, respectively. (**d**,**e** and **f**) were measured imaginary parts of dielectric constant corresponding to (**a**,**b** and **c**), respectively.

Dielectric property is one of the most important characteristics of ferroelectric thin film, which is closely related with electric field^34^. In order to clarify the effect of external field on the ferroelectric superlattice, we extracted permittivity of the ferroelectric superlattices in the two samples^35^. Figures 3 and 4 showed the complex permittivity ɛ of the ferroelectric superlattice films at frequencies ranging from 0.2 THz to 1 THz (6.6 cm^−1^ to 33 cm^−1^) with different external optical powers at room temperature. From Figs 3 and 4, it could be found when without green light illumination, the electric field enhanced the dielectric property and decreased the dielectric loss of the two ferroelectric superlattices. In addition, for the illuminated sample A depicted in Fig. 3, the real part of permittivity was decreased and the imaginary part was increased with the electric voltages from −40 V to 40 V, no matter what the optical power was 200 mW or 400 mW. However, for the illuminated sample B, when the applied optical power was 200 mW, the real part of the permittivity decreased with the electric voltages from −40 V to 40 V. When the optical power was further tuned to 400 mW, the real part of permittivity was increased with the electric voltages from −40 V to 40 V. Besides, negative dielectric constant of sample B was observed, probably because much terahertz wave was reflected. Compared between Figs 3 and 4, it could be found that optical field enhanced the dielectric loss for the two ferroelectric superlattices, leading to a decrease of transmission with optical powers. In addition, electric field played different roles in the dielectric loss of the two illuminated samples, which led to different transmission properties. For illuminated ferroelectric superlattice in the sample A, dielectric loss was increased with external electric voltages varied from −40 V to 40 V. However, for illuminated ferroelectric superlattice in the sample B, variation of dielectric loss with external electric voltages was opposite to that for illuminated sample A.

**Figure 5:**
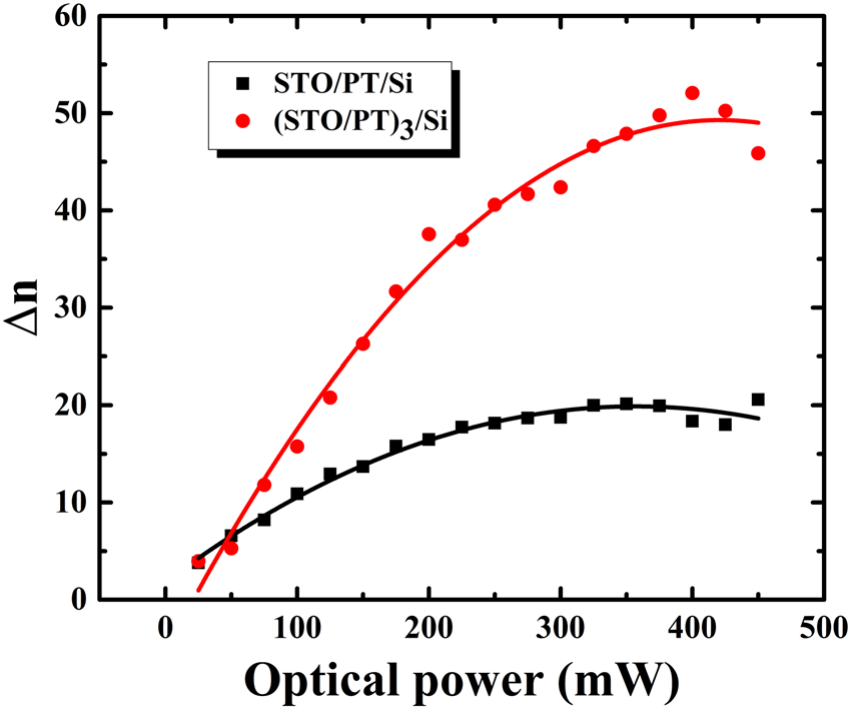
Variation of refractive index dependent on the optical power at 0.4 THz for the three thin films samples. Symbols represent measured data, solid lines are the fits by using Eq. (1).

In order to further illustrate the micro-mechanisms about transmission and dielectric properties in the two different samples, it is worthwhile to study the variation of refractive index under the appearance of external optical fields. Figure 5 displayed the variation of refractive index ${\rm{\Delta }}n$ of the two ferroelectric superlattices with external optical pump. It could be observed that ${\rm{\Delta }}n$ is proportional to the optical powers, in accordance with the relationship Eq. (1) described^36^:

1 $$\begin{array}{rcl}{\rm{\Delta }}n\,(I) & = & -\frac{1}{2}{{\rm{n}}}^{3}{{\rm{r}}}_{eff}{{\rm{E}}}_{sc},\\ {{\rm{E}}}_{{\rm{sc}}} & = & -{E}_{p}(1-\frac{1}{1+I/{I}_{d}}).\end{array}$$

In Eq. (1), r_*eff*_ is the linear electro-optical coefficient. n is the refractive of index. E_sc_ is the space charge field, ${{I}}_{{\rm{d}}}=\beta /s$ is the coefficient of dark irradiation, related with thermal emission rate β and photo excitation area *s*. Besides, the space charge field ${{E}}_{p}=\frac{k\gamma {N}_{{\rm{A}}}}{e\mu }$, where k is the Glass coefficient, γ is the capture area, N_A_ is the acceptor concentration, e is the electrons charge and μ is immigration rate of electrons. When the films were illuminated by the green laser, the external optical field drove the electrons in Ti^3+^ ions to hop to the conduction band, migrate toward a preferred direction in dark areas, and be captured by the trap level of Ti^4+^ ions. In addition, Si substrate was also served as a rich source of carriers. When the optical field was applied on the samples, the excited carriers would immigrate from Si substrate to the ferroelectric thin film due to the concentration difference^37^. From Fig. 5. it could be inferred the larger optical power was tuned, the more carriers were excited. The excited carriers were captured by the traps and then separated into positive and negative charges. The separated charges would form a built-in electric field (the space charge field). But eventually, the variation of refractive would tend to a fixed value as no more carrier could be captured. Compared with sample A, the ferroelectric superlattice in sample B had the larger space charge field. That is because it had more interfaces and more defects, resulting in a larger value of the maximum space charge field. The space charge field could change the refractive index of ferroelectric superlattice due to their excellent electro-optic property.

**Figure 6:**
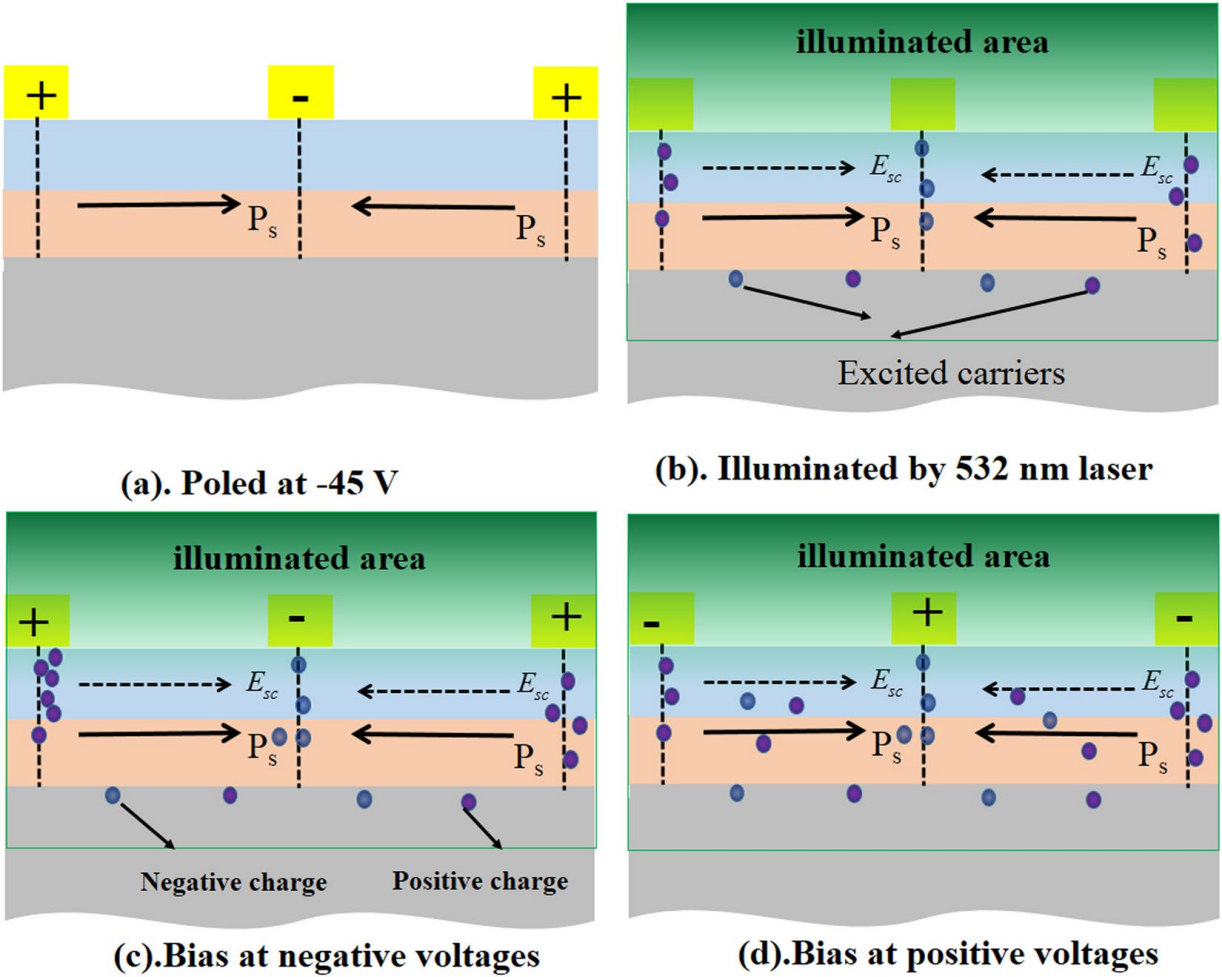
Schematic diagrams to show movement of space charges in sample A with different electric field direction. (**a**) Direction of polarization of poled ferroelectric superlattice in sample A, P_s_ represented the spontaneous polarization; (**b**) Formation of space charge field in the sample A after illumination, E_sc_ is the original space charge field formed by the built-in electric field. (**c**) Movement of carriers in the ferroelectric superlattice with the negative voltages. (**d**) Movement of carriers in the ferroelectric superlattice with the positive voltages.

When the ferroelectric superlattices were applied with the external electric field, which would make contribution to the diffusion and drift of electrons, as shown in Fig. 6. Figure 6 demonstrated the schematic diagrams about the movement of space charges in sample A with different external electric field direction. The space charge field was modified and expressed as follows^38^:

2 $${{E}^{\prime} }_{{sc}}={E}_{0}\frac{{I}_{d}}{I+{I}_{d}}-\frac{{k}_{B}T}{{e}}\frac{d}{dx}\,{ln}(I+{I}_{d})+{E}_{sc},$$

where E_0_ is the applied external electric field, k_B_ is the Boltzmann constant, T is the temperature of the ferroelectric thin film. ${{E}}_{{sc}}^{\prime}$ is the modified space charge field after applying the external electric field, and E_sc_ is the original space charge field without external electric field. So the modified space charge field was intimately related with the direction of external electric field and original space charge field.

From Fig. 2(b), it could be found that the applied positive/negative electric fields had less effect on the transmission property of nonilluminated sample A and displayed a ‘diode’ property in the transmission spectra for the illuminated sample A. It could be found in Fig. 6(b) when the poled ferroelectric superlattice was illuminated by the green laser, original space charge field would have the same direction as the direction of polarization. While ferroelectric superlattice was bias at negative voltages, it would drive negative charges to gather around the positive interdigital electrodes and positive charges gather around the negative charges shown in Fig. 6(c). Such a process led to lower density of electrons in the ferroelectric superlattice between electrodes, which would allow more terahertz wave to transmit. When the ferroelectric superlattice was applied with a positive voltage, the direction of the electric field would be opposite to the polarization. So, the positive electric field would try to reverse the polarization field, which could weaken the space charge field. This process resulted in more electrons diffusing in the ferroelectric superlattice between the electrodes. Consequently, little terahertz wave could transmit through the sample. In addition, much carriers gathered around the electrodes, shielding the effect of positive/negative electric field on the ferroelectric superlattice. Thus, the sample displayed a ‘diode’ property in the transmission spectra. However, ferroelectric superlattice in sample B was thicker than that in sample A and it would lead to less light absorbed by the Si substrate. So excited carriers in the sample B could not shield the effect of electric field effectively and thus the electric field could modulate the transmission of the ferroelectric superlattice. Hence, the transmission was decreased with the increase of the electric voltages, as shown in Fig. 2(c).

**Figure 7:**
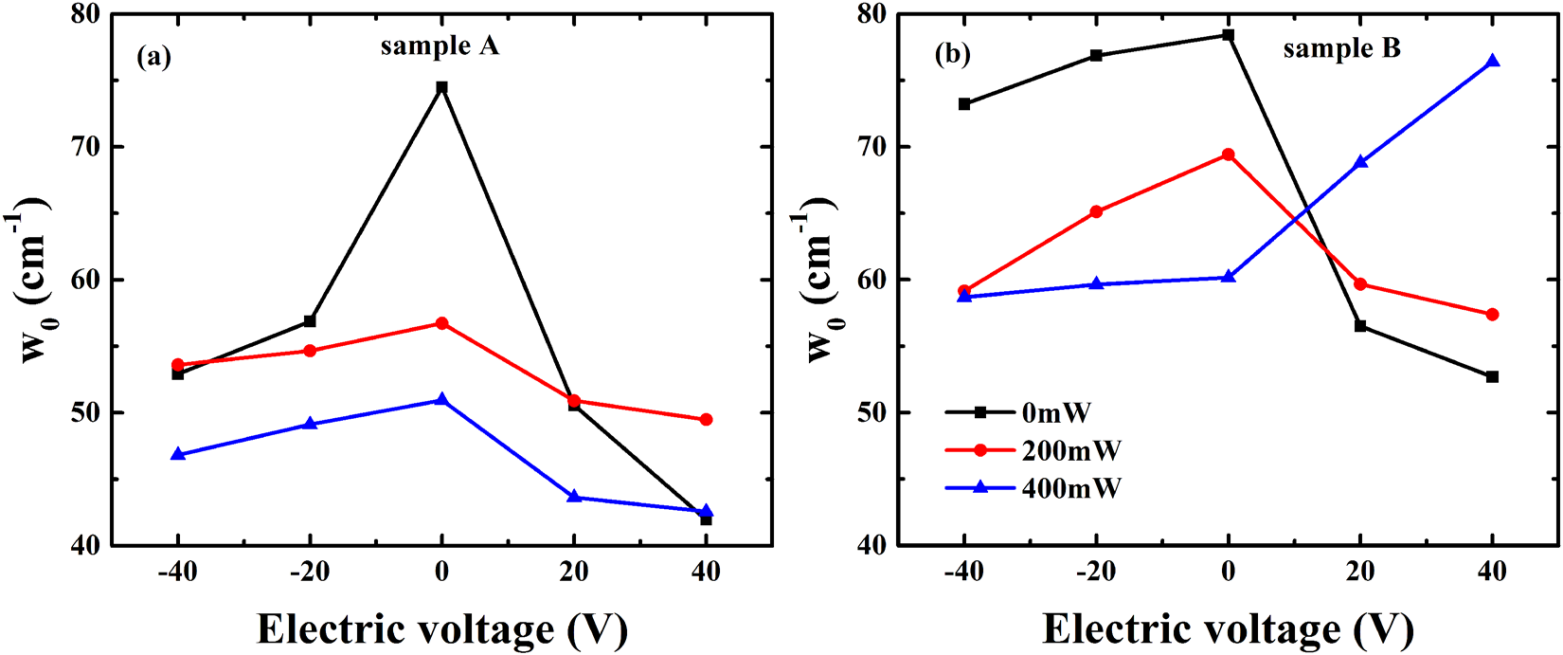
Plots of extracted parameter w_0_ of the two ferroelectric superlattices as function of electric voltages. The black square represented the data for the ferroelectric superlattice without optical pump, the red circle represented the data with the optical power P = 200 mW, the blue triangle represented data with the optical power P = 400 mW.

The external field did not only take effect on the carriers in the ferroelectric superlattices, but also play some roles in the dynamics of ferroelectric superlattice. Relaxation was observed in the lower frequency domain of the dielectric spectra in Figs 3 and 4; this phenomenon might be caused by the domain wall and polar clusters in the strain-induced ferroelectric phase^39,40^. Thus, a more general formula was applied to analyze the THz dielectric spectra. This formula describes the coexistence of Debye relaxation (the central mode) and the Lorentz mode (the soft mode)^31^:

3 $$\varepsilon (\omega )={\varepsilon }_{\infty }+\frac{f(1-{\rm{i}}\omega /\gamma )+{\rm{g}}({\omega }_{0}^{2}-{\omega }^{2}-{\rm{i}}\omega {\rm{\Gamma }})+2\delta \sqrt{fg}}{({\omega }_{0}^{2}-{\omega }^{2}-{\rm{i}}\omega {\rm{\Gamma }})(1-{\rm{i}}\omega /\gamma )-{\delta }^{2}},$$

where ε_∞_ is the high-frequency permittivity; this parameter is the cumulative result of different types of excitation, such as that of electrons, and its value was fixed to 6.37 during the fittings^41,42^. Γ, *f*, and *w*_0_ are the damping coefficient, oscillator strength, and eigenfrequency of the soft mode, respectively. The central model is modeled by Debye relaxation with frequency γ and strength g; δ is a coupling constant. The parameters (*f*, Г, γ) were dependent on the temperature. They were varied little with the external electric voltage, but increased with the external optical powers. As the eigenfrequency of soft mode was related with lattice dynamics, the parameter w_0_ of Eq. (3) were extracted as shown in Fig. 7. The variation of the soft mode involved a polar displacement of the Ti ion away from the central position of the octahedron formed by the neighboring oxygen ions. The amplitude of atomic displacement *u*_*a*_ could be expressed as: ${u}_{a}=\frac{\hslash }{2{\mu }_{0}{w}_{0}}(1+2{n}_{B})$, which in the harmonic approximation is related to the effective mass of the atoms μ_0_, the eigenfrequency of the soft mode w_0_, and the Bose-Einstein factor ${n}_{B}={({e}^{\hslash w/{k}_{B}T}-1)}^{-1}$. It could be inferred for the sample A, the soft mode of the ferroelectric thin film was 74.5 cm^−1^ when without any external field, and it was softened with the increasing values of the electric field. What’s more, optical field also weakened the soft mode of ferroelectric superlattice in sample A due to the built-in electric field. In hybrid fields, the soft mode of ferroelectric superlattice in sample A was also decreased with the external electric field, because diffusion and drift of carriers under the hybrid effects resulted in a larger built-in electric field, leading to the softening of soft mode. Besides, for the ferroelectric superlattice in the sample B, the eigenfrequency of the soft mode was decreased with increase of the electric voltages when the optical power P = 0 mW and 200 mW. Due to the softening of soft mode in ferroelectric superlattice, value of the atomic displacement *u*_*a*_ would get smaller with increasing optical powers and electric voltages, which indicated that lattice was driven to a more asymmetrical structure.

However, when the optical power was tuned to 400 mW, the eigenfrequency of ferroelectric superlattice in sample B was increased from the electric voltages from 0 V to 40 V, and the soft mode was hardened. That is because more electrons were excited and the built-in electric field become larger, which led to a decrease in the total electric field (${\vec{E}}_{0}+{\vec{E}}_{sc}^{\text{'}}$) with the increasing electric voltages. The soft mode was hardened with the positive electric voltages, indicating that the lattice was driven to a more symmetrical structure. The polarization of the ferroelectric superlattice was varied with the modification of the structure, leading to a variation in the dielectric property^43^.

## Conclusion

We compared the transmission and dielectric spectra of two different ferroelectric superlattices on Si substrate under hybrid external fields. When only with optical field, the sample B((STO/PT)3/Si) was more sensitive to the optical field due to more excited defects and more strains in the ferroelectric superlattice. As light was transmitted through the thin film, the two samples displayed entirely different results in transmission. The sample A ((STO/PT)/Si) displayed diode characteristics when with electric field and optical field simultaneously, because of the photorefractive effect and the shielding effects. But for the sample B, the electric voltage could realize a continuous modulation because the sample B with larger thickness ferroelectric thin film had less excited carriers jumped from Si substrate. In addition, the dielectric property of the ferroelectric thin under hybrid fields was also investigated. The optical field changed the relationship between the permittivity and the electric field and enhanced the dielectric loss. Migration of excited carriers caused by the external optical field formed a built-in electric field (space charge field), resulting in variation of the refractive index and dielectric constant. External electric field would take some effect on the original space charge field due to the drift and diffuse of electrons. Furthermore, the optical field and electric field play important roles in the lattice dynamics. The increase of total electric field resulted in softening of the soft mode, which indicated that lattice was driven to a more asymmetrical structure. The results of the present experiment provide a reference for further development of tunable structures controlled by hybrid fields.

## Methods

A radio frequency magnetron sputtering apparatus was employed to prepare the heterostructures ferroelectric thin films. (100)-oriented 20 × 20 × 0.5 mm^3^ Si crystal was used as a substrate. Two different ferroelectric thin films were prepared as below shown in Fig. 1(a): one is a layer of PTO ferroelectric thin film and one layer of STO ferroelectric thin film, the total thickness of the ferroelectric multilayer thin film was 71.9 nm (sample A); Another is an alteration growth of PTO single layer and STO single layer ferroelectric thin film by three cycles, the total thickness of the ferroelectric multilayer thickness was 90.8 nm (sample B). The surface morphology and roughness of the thin film was measured by atomic force microscope (AFM) in contact mode before the preparation of the interdigital electrodes. The surface morphology was very smooth without the formation of visible particles, and the root-mean-square roughness was determined to be lower than 0.5 nm. In sequence, interdigital electrodes consisting of 5-um-wide gold stripes and 300-um-wide gaps were prepared by lift-off photolithography and subsequent deposition of 5-nm-thick Cr film as the adhesion layer and 80-nm-thick Au film by electron beam evaporation. The samples were prepared as a multilayer structure with interdigital electrodes.

The transmission and dielectric properties of the ferroelectric superlattices on Si substrate in THz frequency domain were obtained by a THz time-domain spectrometer (THz-TDS) system produced by Zomega Terahertz Corporation (USA). The detectable frequency range was from 0.1 THz to 1 THz (3.3 cm^−1^ to 33 cm^−1^). The frequency resolution was 4.5 GHz. An all-solid-state green continuous wave laser (center wavelength, 532 nm) was employed for an external optical pumping in this experiment, the green light was obliquely incident upon the film at 60°. In addition, a direct current voltage was also applied to provide an electric field. In this experiment, the wave vector of the terahertz radiation was perpendicular to the plane of the thin film structure, and the electric field of the terahertz rad + iation was also perpendicular to the electrode fingers, as shown in Fig. 1(b).
